# The effect of Chinese herbal formulas combined with metformin on modulating the gut microbiota in the amelioration of type 2 diabetes mellitus: A systematic review and meta-analysis

**DOI:** 10.3389/fendo.2022.927959

**Published:** 2022-09-15

**Authors:** Yunxi Xu, Shuyu Zheng, Shui Jiang, Junyu Chen, Xiaofang Zhu, Ya Zhang

**Affiliations:** Hospital of Chengdu University of Traditional Chinese Medicine, Chengdu University of Traditional Chinese Medicine, Chengdu, China

**Keywords:** type 2 diabetes mellitus, gut microbiota, metformin, Chinese herbal formula, combination treatment, meta-analysis

## Abstract

To assess and analyse the effectiveness and safety of combined Chinese herbal formula (CHF) and metformin treatment in the modulation of the gut microbiota in the amelioration of type 2 diabetes mellitus(T2DM), all publications addressing the effect of this combination treatment on the quantitative alterations in the gut microbiota and glucose parameters were collected. Rob tool in the Cochrane handbook was performed to evaluate the methodological quality of all included studies. Relevant information and statistics were abstracted and synthesized in Review Manager 5.4 to evaluate the efficacy of combination treatment. Sensitivity analyses and subgroup analyses were used to analyse the sources of heterogeneity. Publication bias analyses were performed by Stata software to assess the robustness and quality of the outcomes. As a result, a total of 12 eligible RCTs with 1307 T2DM participants from 7 electronic databases were included. Combined CHF with metformin treatment showed better efficacies than metformin monotherapy in regulating the structure of the gut microbiota, characterized by increased Bifidobacterium, Lactobacillus and Bacteroidetes and decreased Enterobacteriaceae, Enterococcus, and Saccharomyces along with better decreases in glycated haemoglobin, fasting plasma glucose, 2-hour postprandial blood glucose, fasting insulin and homeostasis model assessment of insulin resistance. Subgroup analyses further analysed the effect of metformin doses and CHF classifications on controlling hyperglycaemia and altering the gut microbiota. In conclusion, our meta-analysis suggested that combined CHF with metformin treatment is promising for the modulation of the gut microbiota along with ameliorating hyperglycemia in T2DM patients. Importantly, more well-designed RCTs are needed to validate the outcomes and verify the treatment value for clinical purposes.

**Systematic Review Registration:**

https://www.crd.york.ac.uk/prospero/display_record.php?ID=CRD42021291524, identifier CRD42021291524.

## 1 Introduction

The number of people affected by type 2 diabetes mellitus (T2DM), a disease with worldwide prevalence characterized by elevated blood glucose levels (hyperglycaemia) (1, 2), is estimated to increase to more than 700 million people by 2045 (3). Pharmacotherapies, such as oral hypoglycemic agents(metformin, glimepiride, pioglitazone and Acarbose) and insulin injections, and other regimens, such as weight control and diet management, are suggested for T2DM patients (4). Despite the availability of various antidiabetic treatments, current antidiabetic medicines fail to achieve satisfactory hypoglycaemic effects in all patients, and some adverse reactions, such as gastrointestinal disturbances, can occur (5). Thus, further attempts to develop more efficient and safer therapeutic methods for T2DM to achieve satisfactory outcomes are urgently needed.

The gut microbiota is considered as a salient vehicle emerging in the pathogenesis and development of metabolic diseases. (6) The gut microbiota is formed by up to 100 trillion microorganisms, including bacteria, viruses, fungi, and protozoa (7), among which the majority are bacteria (8). An accumulating number of pivotal reports have demonstrated that the gut microbiota serves as a novel contributory factor in digestion, metabolism, pathogen defence and immunity (9). The core role of the gut microbiota in metabolic diseases originates from the realization that the gut microbiota can trigger the inflammatory reaction mediator lipopolysaccharide(LPS) to induce insulin resistance (10). The low-grade inflammation reaction, as an important pathophysiological factor resulting in hyperglycaemia and insulin resistance (11), can be activated by an increase in the permeability of gut microbiota and a reduction in the intestinal barrier function which transfers Gram-negative bacteria into the systemic circulation and elevates the LPS levels (12). The evidence that the dysbiosis has existed in the intestinal tract of T2DM patients and the oral administration of prebiotic such as Lactobacillus contributes to ameliorating hyperglycaemia and alleviating endotoxemia (6, 12) further validates that the dysbiosis of gut microbiota could disturb the integrity of the gut barrier and reshape the host metabolism, implicating in the susceptibility to the onset and progression of insulin resistance (13, 14).

Microbial-mediated therapeutic strategies are being sought to treat T2DM (8). Metformin, a widely applied oral anti-hyperglycaemic agent, exerts its hypoglycaemic action mainly by inhibiting hepatic gluconeogenesis and increasing the utilization of glucose in peripheral tissues (15). Evidence suggested that the bioavailability of metformin was higher in the intestinal tract than in the serum (16). More trials further revealed microbial changes along with the promotion of glucose metabolism in metformin treatment groups (17). In parallel, Chinese herbal formulas (CHFs), with their unique theory and accumulated considerable clinical experience (18, 19), have been concluded to have remarkable therapeutic effects on diabetes, with fewer side effects than metformin (20). Significant reductions in glycated haemoglobin(HbA1c), fasting plasma glucose(FPG), 2-hour postprandial blood glucose(2hPG), fasting insulin(FINS) and homeostasis model assessment of insulin resistance(HOMA-IR) have been found after treatment with CHFs (18, 21, 22). Early intervention with CHFs can delay diabetes-related macrovascular complications by regulating the AMPK signalling pathway (23). A recent study showed that a CHF decoction exerted beneficial effects on diabetic hindlimb ischaemia by reshaping the gut microbiota structure and stunning the VEGF/HIF-1α pathway (24). In addition, Zheng Y showed that CHFs could modulate the quantity of probiotic and pathogenic bacteria, which was accompanied by the control of blood glucose and insulin resistance at multiple levels (25), which suggested that both metformin and CHFs can be used in the treatment of diabetes mellitus by modulating gut microbiota dysbiosis.

Encouragingly, recent studies introduced the concept that metformin combined with CHFs had synergistic effects to improve the clinical efficacy of antidiabetic treatment for improving glucose and lipid metabolism through multiple pathways in line with reducing the associated adverse reactions (26, 27). Huangkui capsules combined with metformin effectively improved diabetic nephropathy *via* the Klotho/TGF-β1/p38 MAPK signalling pathway (28). The application of Shenqi Jiangtang granules with metformin effectively controlled blood glucose and lipid levels, enhanced antioxidant capacity, and reduced the levels of inflammatory cytokines in gestational diabetes mellitus. (29) More studies further confirmed that combined treatment with CHFs and metformin had significant additive and synergistic effects on the changes in the gut microbiota, such as increased Bifidobacterium, Bacteroides, and Lactobacillus and decreased Enterobacter and Enterococcus, compared with metformin monotherapy (30–32), supporting that the gut microbiota can be seen as a promising therapeutic target of combination therapy in the treatment of diabetes. To the best of our knowledge, few systematic and comprehensive studies have investigated the effects of combination treatment on the changes in the gut microbiota in T2DM patients. According to this background, this study aimed to conduct a meta-analysis to evaluate the effectiveness and quality of this combination treatment on the alteration of the gut microbiota in the remission of type 2 diabetes mellitus.

## 2 Materials and methods

This meta-analysis was registered in PROSPERO with the registration number CRD42021291524(Record ID=291524) and was performed in accordance with the instructions of the Preferred Reporting Items for Systematic Reviews and Meta-Analyses (PRISMA) statement (33).

### 2.1 Literature search strategy

Electronic databases, including the PubMed, Web of Science, Cochrane Library, China National Knowledge Infrastructure (CNKI), Wan Fang China, VIP Medical Information, and Chinese Biomedical Literature (CBM) databases, were searched to screen for all relevant published studies on the therapeutic value of CHFs combined with metformin in the treatment of diabetes from database inception to November 2021. The keywords or MeSH terms for the search strategy were “type 2 diabetes mellitus”, “gastrointestinal microbiome” or “gut microbiota” or “intestinal flora”, “traditional Chinese medicine”, and “hypoglycemic agents” within the English and Chinese languages.

### 2.2 Eligibility criteria

All randomized controlled trials meeting the following eligibility criteria were included:

- Participants: studies that enrolled patients who were diagnosed with type 2 diabetes mellitus.
- Intervention: studies that used treatment with CHFs and metformin in the experimental group.
- Comparison: studies that used metformin in the control group.
- Outcomes: studies that investigated the quantitative alterations of the gut microbiota and glucose metabolism indicators. The primary outcome was the quantitative changes in the gut microbiota, especially the statistics for Bifidobacterium, Lactobacillus, Bacteroidetes Enterobacteriaceae, Enterococcus, and Saccharomyces. The secondary outcome was the quantitative changes in glucose metabolism indicators, including fasting plasma glucose (FPG), 2-hour postprandial blood glucose (2hPG), glycosylated haemoglobin (HbA1c), fasting insulin (FINS) and homeostasis model assessment of insulin resistance (HOMA-IR). All outcomes presented as continuous variables were included in the meta-analysis.

### 2.3 Exclusion criteria

Studies meeting any of the following exclusion criteria were excluded:

- Studies that did not conform to this research topic, as well as studies that were case reports, animal studies, reviews and repeated publications;
- Studies that did not include type 2 diabetes mellitus patients in the experiment or enrolled type 1 diabetes mellitus patients in the trial;
- Studies that did not use treatment with CHFs and metformin in the experimental group;
- Studies that did not use metformin in the control group or used insulin as a control intervention.
- Studies that did not provide explicit statistics for changes in the gut microbiota, especially those in which statistics were not presented in the form of the mean ± standard deviation.

### 2.4 Study selection and data extraction

The electronic databases were independently searched and screened by two reviewers (Shui Jiang and Junyu Chen) according to the inclusion and exclusion criteria. Two authors (Yunxi Xu and Shuyu Zheng) extracted data and collected the data from the included studies. The extracted information included the first author, publication year, participants, intervention measures of the experimental group and control group, information on the included formulas, data on the gut microbiota and glucose metabolism indicators in the experimental and control groups, course of treatment and adverse reactions. If any disagreement occurred, a senior reviewer (Ya Zhang) was consulted to assist in the discussion.

### 2.5 Methodological evaluation

The methodological quality evaluation of each included study was performed by two authors (Shuyu Zheng and Xiaofang Zhu) through the Cochrane Collaboration risk of bias (Rob) tool (34) from six aspects: selection bias (random sequence generation, allocation concealment), performance bias, detection bias, attrition bias, reporting bias and other biases. Each item was judged as “low”, “high”, or “unclear risk of bias” to assess the risk of bias of each study. Any discrepancies were resolved and a consensus was reached by discussion between the two authors.

### 2.6 Data synthesis and analyses

Relevant information and statistics were synthesized by Review Manager 5.4 to evaluate the efficacy of the combination treatment. Heterogeneity tests were conducted for every outcome. If the heterogeneity was not statistically significant (I^2^ <50%), a fixed effect model was used for meta-analysis to analyse the outcome; if the heterogeneity was statistically significant (I^2^ >50%), a random effect model was used for meta-analysis. To explore the source of heterogeneity, sensitivity analysis was used to clarify the impact of individual trials on the overall outcome. If sensitivity analysis suggested that the impact on the overall outcome was stable, subgroup analysis was further planned to explore the sources of the heterogeneity.

### 2.7 Publication bias

To assess possible publication biases in the analysed outcomes, Begg’s funnel plots (with pseudo 95% confidence limits) and Egger’s publication bias plots were employed by observing the symmetry of these plots formed by Stata software. Additionally, Begg’s test and Egger’s test can assess publication bias in a quantitative manner. Pr > |z| in Begg’s test and Pr > |t| in Egger’s test >0.05 indicated no obvious publication bias in the analysed outcomes (35).

## 3 Results

### 3.1 Literature search

**Figure 1:**
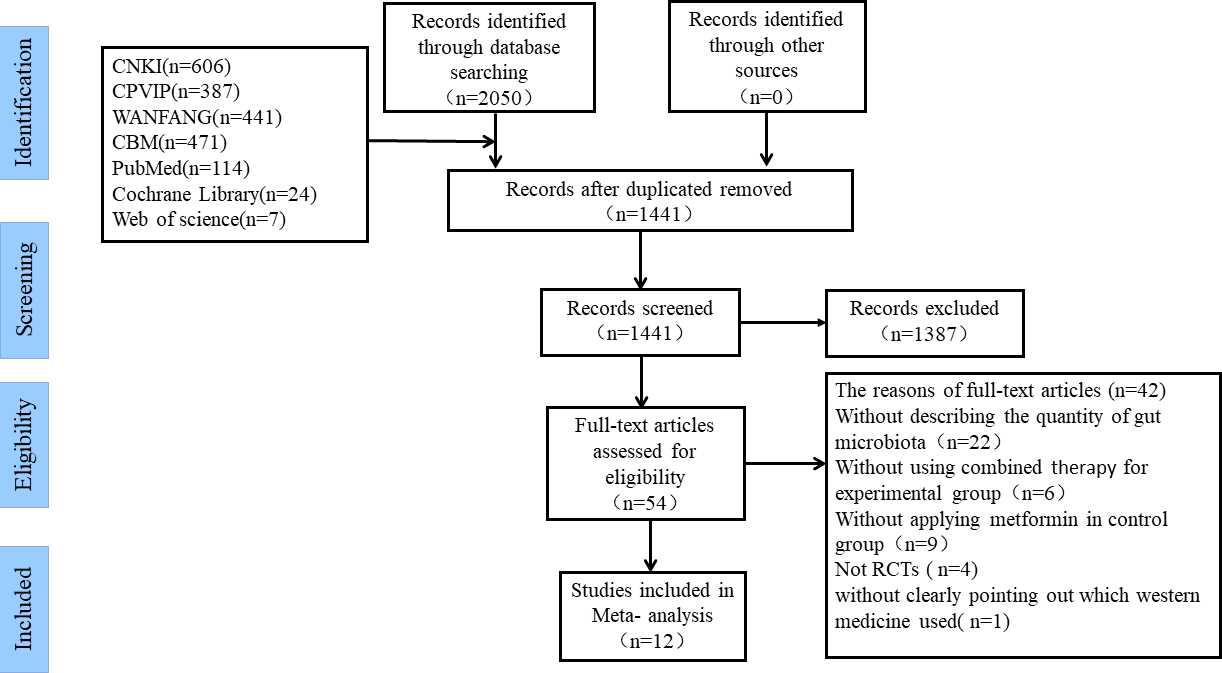
The inclusion and exclusion process.

By screening 7 electronic databases, 2050 studies potentially eligible for this study were initially obtained. Then, after duplicated articles were removed, 1441 studies remained. Of the 1441 studies, 1387 irrelevant studies were excluded by reviewing the titles and abstracts. Afterwards, the remaining 54 studies were assessed according to the inclusion and exclusion criteria by carefully reading the full text. Ultimately, 12 studies were confirmed to be eligible for inclusion in this meta-analysis (36–47). The inclusion and exclusion process for each selected study is shown in **Figure 1**. 

### 3.2 Main characteristics of the studies and interventions

The detailed characteristics of the 12 included studies are summarized in **Table 1**. The 12 studies (36–47), which enrolled a total of 1307 T2DM patients (653 in experimental groups and 654 in control groups), were all RCTs and were reported in the Chinese language. Combined CHF and metformin interventions were applied in the experimental groups of the 12 studies. Metformin was used in the control groups of the 12 studies. The studies all compared the effects of combined CHF and metformin treatment and those of metformin monotherapy on the alterations of the gut microbiota in T2DM patients. Notably, the changes in glucose metabolism indicators were also analysed in 10 trials (36–38, 40–42, 44–47). The doses of metformin used varied in the 12 studies, which were mainly divided into two drug doses: 0.25 g and 0.5 g. Detailed information on metformin is summarized in **Table 2**. The fundamental therapeutic principles of the CHFs used in the experimental groups of 9 studies were characterized by clearing the heat and excreting dampness and blood stasis. The fundamental therapeutic principle of the CHFs used in the remaining 3 studies was tonification, mainly by invigorating the viscera and replenishing qi and yang. The CHF that was most frequently utilized among the included studies was Gegen Qinlian decoction (36, 45, 46). The fundamental therapeutic principles and compositions of the formulas used in the experimental groups are shown in **Table 3**. The beneficial effects of CHFs combined with metformin on the amelioration on host metabolism, modulation of gut microbiota, promotion of symptoms/therapeutic efficiency and safety evaluation in type 2 diabetic patients in detail are summarized in **Table 4**.

**Table 1:** Detailed characteristics of the eligible studies.

Included studies	Groups		Intervention measures		Outcomes	Course of treatment	Adverse reaction
	Experimental group	Control group	Experimental group	Control group		
Chen Qiao,2021 (36)	32	32	Gegen Qinlian decoction+metformin	Metformin	1,2,4,5,7,8	1 month	not mentioned
Jiang Haiyan,2021 (37)	42	42	Shenlin Baizhu Powder+metformin	Metformin	1,3,5,9	6 month	reported
Tang Anna,2021 (38)	25	20	Jinmai Wendan Decoction+OADs	Metformin	1,2,3,4,5,7,8,9	12 weeks	not reported
Zhang Hao,2020 (39)	42	42	Maren Wan+metformin	Metformin	1,2,3,4,5,6	3 month	not mentioned
Liu Yadong,2020 (40)	100	100	Qingfei Xiegan decoction+metformin	Metformin	1,2,3,4,5,6,7,8,10,11	2 month	reported
Liu Xiaoxi,2019 (41)	48	47	Tonifying Qi and Strengthening Spleen decoction+metformin	Metformin	1,2,3 ,4,5,6,7,10,11	2 month	not mentioned
Dai Congshu,2019 (42)	67	65	Qingfei Xiegan decoction+metformin	Metformin	1,2,3,4,5,6,7,8,9,10,11	2 month	reported
Li Yancan,2019 (43)	129	128	Qingre huoxue huatan formula+metformin	Metformin	1,2,3,4,5,6	3 month	reported
Chen Zhongshan,2019 (44)	21	21	Jinmai Wendan decoction+OADs	Metformin	1,2,3,4,5,7,8,9,10	12weeks	not reported
Li Hua,2018 (45)	48	48	Compound Gegen Qinlian decoction+metformin	Metformin	1,4,7,8,9	2 week	not mentioned
FENG Xin-ge,2016 (46)	50	60	Gegen Qinlian decoction+metformin	Metformin	1,4,7,8,9	3 month	not reported
Li Jiwu ,2015 (47)	49	49	Wenyang Yiqi Huoxue Formula+metformin	Metformin	1,2,3,4,5,6,10,11	2 month	not mentioned
1,Bifidobacterium	2,Lactobacillus	3,Bacteroidetes	4,Enterobacteriaceae	5,Enterococcus	6,Saccharomycetes	7,FPG	8,2hPG
9,HbA1c%	10,FINS	11,HOMA-IR					

**Table 2:** Detailed information on metformin.

Included studies	Specifications of metformin		Manufacturer	NMPN	Batch number
	Expeimental group	Control group			
Chen Qiao,2021 (36)	0.75 g	0.25 g	GUANGZHOU BAIYUNSHAN TIANXIN PHARMACEUTIACL CO.,LTD.	H44023514	not given
Jiang Haiyan,2021 (37)	0.5g	0.5g	CHANGZHOU PHARMACEUTIACL FACTORY CO.,LTD.	H20054786	not given
Tang Anna,2021 (38)	0.25g	0.25g	CHANGZHOU PHARMACEUTIACL FACTORY CO.,LTD.	H20054786	not given
Zhang Hao,2020 (39)	0.5g	0.5g	HANSON PHARMA	H20143248	not given
Liu Yadong,2020 (40)	0.5g	0.5g	SINO-AMERICAN SHANGHAI SQUIBB PHARMACEUTICALS LTD.	not given	20151108
Liu Xiaoxi,2019 (41)	0.25 g	0.25 g	not given	not given	not given
Dai Congshu,2019 (42)	0.25 g	0.25 g	Jilin Jinheng Pharmaceutical Co., Ltd.	H22023147	not given
Li Yan-can,2019 (43)	0.5 g	0.5 g	SINO-AMERICAN SHANGHAI SQUIBB PHARMACEUTICALS LTD	H20023370	not given
Chen Zhongshan,2019 (44)	0.25g	0.25g	CHANGZHOU PHARMACEUTIACL FACTORY CO.,LTD.	H20054786	not given
Li Hua,2018 (45)	0.5g	0.5 g	not given	not given	not given
FENG Xin-ge,2016 (46)	0.5g	0.5g	Shanghai pharmaceutical Sine CO.,LTD.	not given	59131112
Li Jiwu,2015 (47)	0.25g	0.25g	SUZHONG PHARMAR	H32021625	not given

NMPN, National Medicine Permission Number.

**Table 3:** The therapeutic principles and compositions of the CHFs in the experimental groups.

Included studies	Fundmental theraputic principles	Name of CHFs	CHFs composition / manufacturer	Latin name of the herbs in CHFs
Chen Qiao,2021 (36)	Clearing the internal heat,drying dampness and detoxification	Gegen Qinlian decoction	Gegen 15g, Huangqin 9g, Huanglian 9g, Gancao 6g	Radix pueraria 20g, Coptidis Rhizoma12g, Scutellariae Radix12g, Glycyrrhizae Radix 6g
Jiang Haiyan,2021 (37)	Invigorating the spleen and replenishing qi, excreting dampness and stopping diarrhea	Shenlin Baizhu powder	Beijing Tongrentang Pharmaceutical Factory, national medicine standard number Z1102075 (detailed drugs and dosages are not given)	//
Tang Anna,2021 (38)	dissipating phlegm and removing blood stasis	Jinmai Wentan decoction	Gualou 15g, Danshen 10g,Bnaxia 10g,Houpo 10g,Zhuru 10g,Yujin 10g,Maidong 10g,Zheshi 10g	Fructus trichosanthis 15g, Radix salviae Miltiorrhizae 10g, Rhizoma pinelliae 10g, Cortex magnoloae Officinalis 10g, Caulis bambusae In Taenia 10g, Radix curcumae 10g, Radix ophiopogonis 10g, Haematitum 10g
Zhang Hao,2020 (39)	Moistening the intestines and clearing heat, promoting qi and relieving defecation	Maren Wan	Huomaren, Dahuang, Baishao, Kuxingren, Houpou, Zheshi (Nanjing Tongrentang Pharmaceutical Co., Ltd., Z32020097, the detailed dosages are not given)	Fructus cannabis, Radix Et Rhizoma Rhei, Radix paeoniae Alba, Semen armeniacae Amarum, Cortex magnoloae Officinalis, Haematitum
Liu Yadong,2020 (40)	Clearing heat and detoxification, resolving turbidity and excreting dampness	Qingfei Xiegan decoction	Gaoben 15 g, Baizhi 15 g, Huangqin 15 g, Wannianhao 15 g, Gegen 20 g, Laifuzi 30 g, Tufuling 20 g, Pugongying 20 g,, Jiegeng 10 g, Shengma 10 g, Dahuang 5 g	Rhizoma ligustici 15g, Radix angelicae Dahuricae 15g, Scutellariae Radix 15g, Artemisia gmelinii Web ex Stechm 15g , Radix pueraria 20g, Semen raphani 30g, Rhizoma smilacis Glabrae 20g, Herba taraxact 20g, Radix platycodonis 10g, Rhizoma cimicifugae 10g, Radix et rhizoma Rhei 5g
Liu Xiaoxi,2019 (41)	Replenishing qi and invigorating the spleen, promoting body fluidto quench thirst	Tonifying Qi and strengthening spleen decoction	Liaoning College of traditional Chinese Medicine Pharmaceutical Co., Ltd., approval number: national medicine Z21020756 (detailed drugs and dosages are not given)	//
Dai Congshu,2019 (42)	Clearing heat and detoxification, resolving turbidity and excreting dampness	Qingfei Xiegan decoction	Gegen 20 g, Gaoben 15 g, Shengma 10 g, Baizhi 15 g, Huangqin 15 g, Jiegeng 10 g, Laifuzu 30 g, Dahuang 5 g(decocted latter),Tufuling 20 g, Pugongying 20 g, Wannianhao 15 g	Radix pueraria 20g, Rhizoma ligustici 15g, Rhizoma cimicifugae 10g, Radix angelicae Dahuricae 15g, Scutellariae Radix 15g, Radix platycodonis 10g, Semen raphani 30g, Radix et rhizoma Rhei 5g, Rhizoma smilacis Glabrae 20g, Herba taraxact 20g, Artemisia gmelinii Web ex Stechm 15g
Li Yancan,2019 (43)	Clearing heat,dissipating phlegm, removing blood stasis	Qingre Huoxue Huatan formula	Huanglian 10g, Huangqin10g, Mudanpi 10g, Taoren 10g, Fuling 10g, Danggui 15g, Chishao 15g, Danshen 15g, Dangshen 15g	Coptidis Rhizoma 10g, Scutellariae Radix 10g, Cortex moutan 10g, Semen persicae 10g, Poria 10g, Radix angelicae Sinensis 15g, Radix paeoniae Rubra 15g, Radix salviae Miltiorrhizae 15g, Radix codonopsis 15g
Chen Zhongshan,2019 (44)	dissipating phlegm and removing blood stasis	Jinmai Wentan decoction	Gualou 15g, Danshen 10g,Bnaxia 10g,Houpo 10g,Zhuru 10g,Yujin 10g,Maidong 10g,Zheshi 10g	Fructus trichosanthis 15g, Radix salviae Miltiorrhizae 10g, Rhizoma pinelliae 10g, Cortex magnoloae Officinalis 10g, Caulis bambusae In Taenia 10g, Radix curcumae 10g, Radix ophiopogonis 10g, Haematitum 10g
Li Hua,2018 (45)	Clearing the internal heat,drying dampness and detoxification	Compound Gegen Qinlian decoction	Gegen 20g, Huanglian 12g,Huangqin 9g,Gancao 6g	Radix pueraria 20g, Coptidis Rhizoma 12g, Scutellariae Radix 12g, Glycyrrhizae Radix 6g
FENG Xin-ge,2016 (46)	Clearing the internal heat,drying dampness and detoxification	Gegen Qinlian decoction	Gegen 20g, Huanglian 12g, Huangqin 9g,Gancao 6g	Radix pueraria 20g, Coptidis Rhizoma 12g, Scutellariae Radix 12g, Glycyrrhizae Radix 6g
Li Jiwu,2015 (47)	Warming yang, invigorating qi and activating blood circulation	Wenyang Yiqi Huoxue formula	Fupian(decoted first) 10g, Renshen 10g, Zhigancao 10g, Baizhu 15g, Fuling 15g Zhike 15g, Chishao 15g, Shanyurou 15g, Beichaihu 15g, Guizhi 8g, Ganjiang 8g, Danshen 20g	Radix aconiti lateralis Preparata 10g, Radix ginseng 10g, Radix glycyrrhizae Preparata 10g, Radix angelicae Dahuricae 15g, Poria 10g, Fructus aurantii 15g, Radix paeoniae Rubra 15g, Fructus corni 15g, Bupleuri Radix 15g ,Ramulus cinnamomi 8g, Rhizoma zingiberis 8g, Radix salviae Miltiorrhizae 20g

**Table 4:** Summary of the beneficial effects of CHFs combined with metformin.

Included studies	Amelioration on host metabolism	Modulation on gut microbiota	Promotion of symptoms/therapeutic efficiency	Safety evaluation
Chen Qiao,2021 (36)	No significant change was observed in the levels of FPG, 2hPG(p>0.05)	Gegen Qinlian decoction+metformin treatment contributed to a lower abundance of Enterobacteriaceae, Enterococcus and led to a higher abundance of Bifidobacterium and lactobacillus than those in the metformin group(p<0.05).	The symptoms of diarrhea, abdominal distension, abdominal pain, and epigastric fullness were alleviated after treated by Gegen Qinlian decoction+metformin therapy.	Not mentioned
Jiang Haiyan,2021 (37)	The level of FINS was significantly elevated and the levels of FPG and HbA1c were remarkably decreased in the Shenlin Baizhu Powder+metformin group than those in the metformin group(p<0.05).	Shenlin Baizhu Powder+metformin treatment contributed to a more significant abundance of Bifidobacterium and lactobacillus, and led to a lower abundance of Enterococcus (P<0.05).	Not mentioned	In the Shenlin Baizhu Powder+metformin group, constipation was observed in 3 participants, vomiting was observed in 2 participants, fatigue was observed in 2 participants; In the metformin group, constipation was observed in 2 participants, vomiting was observed in 3 patients, fatigue was observed in 4 patients, and impaired hepatic and renal functions were found in 1 patient. (χ^2^=2.59, P=0.63)
Tang Anna,2021 (38)	The levels of 2hPG, HbA1c, TG, and LDL-C in the Jinmai Wendan Decoction+OADs group were significantly lower than those in the OADs group(p<0.05).	Jinmai Wendan Decoction+OADs contributed to a higher abundance of Clostridium and Lactobacillus than metformin used alone(P<0.05).	Not mentioned	Side effects such as Liver and kidney damage were not found in T2DM patients treated by Jinmai Wendan Decoction.
Zhang Hao,2020 (39)	not mentioned	In the Maren Wan+metformin group, probiotic bacteria Bifidobacterium, Lactobacillus and Bacteroidetes were significantly higher than those in the metformin group (P<0.05); Meanwhile, pathogenic bacteria Enterobacteriaceae, Enterococcus, and Saccharomyces were significantly lower than those in the metformin group (P<0.05).	Not mentioned	not mentioned
Liu Yadong,2020 (40)	The levels of FPG, 2hPG, FINS, HOMA-IR, TNF-α, IL-6 were significantly lower in the Qingfei Xiegan decoction+metformin group than those in the metformin group (P < 0.05).	Qingfei Xiegan decoction+metformin group remarkably contributed to a higher abundance of Bifidobacterium, Lactobacillus and Bacteroidetes as well as led to a lower abundance of Enterobacteriaceae, Enterococcus and Saccharomyces than metformin used alone(P < 0.05).	The therapeutic efficiency of the Qingfei Xiegan decoction+metformin treatment was higher than that of the metformin monotherapy (χ2 =5.181, p=0.023, p<0.05).	In the Qingfei Xiegan decoction+metformin group, mild diarrhea was found in 3 patients. In the control group, mild nausea and vomiting were observed in 2 patients, all of which were relieved after symptomatic treatment and did not affect the course of treatment.
Liu Xiaoxi,2019 (41)	TC, TG, FPG, FINS, and HOMA-IR were significantly improved in the Tonifying Qi and Strengthening Spleen decoction+metformin group than those in the metformin group (P < 0. 05).	Tonifying Qi and Strengthening Spleen decoction+metformin treatment remarkably contributed to a higher abundance of Bifidobacterium, Lactobacillus and Bacteroidetes as well as led to a lower abundance of Enterobacteriaceae, Enterococcus and Saccharomyces than metformin used alone(P < 0.05).	The therapeutic efficiency of the Tonifying Qi and Strengthening Spleen decoction+metformin treatment was higher than that of the metformin monotherapy (χ2 =8. 954,P < 0. 05)	not mentioned
Dai Congshu,2019 (42)	The levels of FPG, 2hPG, HbA1c, FINS, HOMA-IR,TC,TG,LDL-C, IL-6, IL-8, TNF-α were notably decreased and HDL-C was significantly elevated in the Qingfei Xiegan decoction+metformin than those in the control group(P < 0.01)	Qingfei Xiegan decoction+metformin treatment remarkably contributed to a higher abundance of Bifidobacterium, Lactobacillus and Bacteroidetes as well as led to a lower abundance of Enterobacteriaceae, Enterococcus and Saccharomyces than metformin used alone(P < 0.05).	The therapeutic efficiency of the Qingfei Xiegan decoction+metformin treatment was higher than that of the metformin monotherapy( Z=2.535, P< 0. 01)	In the Qingfei Xiegan decoction+metformin group, 6 patients were observed gastrointestinal disturbances such as abdominal pain, diarrhea, nausea and vomiting; In the metformin group, 5 patients were observed symptoms of gastrointestinal disturbances.
Li Yancan,2019 (43)	not mentioned	Qingre huoxue huatan formula+metformin remarkably contributed to a higher abundance of Bifidobacterium, Lactobacillus and Bacteroidetes as well as led to a lower abundance of Enterobacteriaceae, Enterococcus and Saccharomyces than metformin used alone(P < 0.05).	The frequency of defecation per week was significantly increased and the defecation time was significantly shortened compared with the control group(P < 0. 05).	There was no significant difference in the incidence of adverse reactions between the two groups (P > 0. 05).
Chen Zhongshan,2019 (44)	The levels of FPG, 0.5hPG, 2hPG, HbA1c, TC, LDL-C were notably decreased in the Jinmai Wendan decoction+OADs group than those in the OADs group(P < 0. 05).	The number of Clostridium and Firmicutes in the Jinmai Wendan decoction+OADs group were significantly higher than those in the OADs group (P < 0.05).	not mentioned	Adverse reactions and impaired hepatic and renal functions were not found in the patients treated by Jinmai Wendan decoction.
Li Hua,2018 (45)	The levels of FBG, 2hPG, HbA1c were notably decreased in the compound Gegen Qinlian decoction+metformin group than those in the metformin group(P < 0. 05).	Compound Gegen Qinlian decoction+metformin contributed to a higher abundance of Clostridium and Bifidobacterium as well as contributed to a lower abundance of Enterobacteriaceae(P < 0.05).	The therapeutic efficiency of the Compound Gegen Qinlian decoction+metformin treatment was higher than that of the metformin monotherapy(P < 0. 05).	not mentioned
FENG Xin-ge,2016 (46)	The levels of FBG, 2hPG, HbA1c were notably decreased in the Gegen Qinlian decoction+metformin group than those in the metformin group(P < 0. 05).	Gegen Qinlian decoction+metformin contributed to a higher abundance of Clostridium and Bifidobacterium as well as contributed to a lower abundance of Enterobacteriaceae(P < 0.05).	The therapeutic efficiency of the Gegen Qinlian decoction+metformin treatment was higher than that of the metformin monotherapy(P < 0. 05).	Adverse reactions were not found in the two groups.
Li Jiwu ,2015 (47)	The levels of FINS, HOMA-IR, TC, TG, LDL-C were notably decreased and HDL-C was significantly elevated in the Wenyang Yiqi Huoxue Formula+metformin group than those in the control group(P < 0. 05).	Wenyang Yiqi Huoxue Formula+metformin contributed to a higher abundance of Bifidobacterium, Lactobacillus, Bacteroidetes than that in the metformin group(P < 0. 05)	The therapeutic efficiency of the Wenyang Yiqi Huoxue Formula+metformin treatment was higher than that of the metformin monotherapy(P < 0. 05).	not mentioned

### 3.3 Methodological quality evaluation

**Figure 2:**
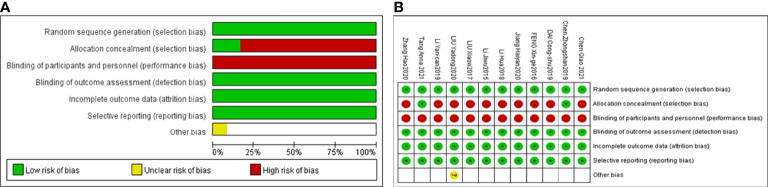
Risk of bias graph **(A)**, risk of bias summary **(B)**.

Twelve included studies followed the principle of randomization, among which 10 studies (36, 38, 40–47) referred to the randomization number method and 2 studies described allocation concealment methods (38, 44). Six included studies explicitly mentioned adverse reactions (37, 38, 40, 42–44). Performance bias information was not mentioned in any of the 12 articles. The detection bias, attribution bias and reporting bias were low for those 12 studies. Information on the follow-up duration was not mentioned in any of the 12 studies. The detailed quality assessments for each study are shown in **Figure 2**.

### 3.4 Meta-analysis results

#### 3.4.1 Probiotic bacteria

To compare the effects of combined CHF and metformin treatment with those of metformin monotherapy on the quantitative changes in probiotic bacteria, 10 studies (36, 37, 39–43, 45–47) evaluating changes in Bifidobacterium, 7 studies (36, 39–43, 47) evaluating changes in Lactobacillus and 7 studies (37, 39–43, 47) evaluating changes in Bacteroidetes as the primary outcome were included in the analyses.

**Figure 3:**
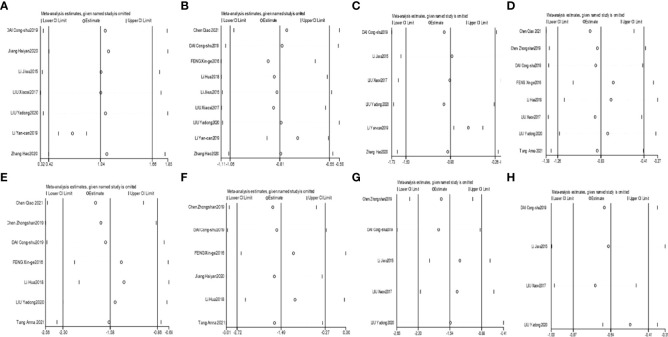
Sensitivity analyses of Bacteroidetes **(A)**, Enterobacteriaceae **(B)**, Saccharomyces **(C)**, FPG **(D)**, 2hPG **(E)**, HbA1c **(F)**, FINS **(G)**, and HOMA-IR **(H)**.

The heterogeneity for Bifidobacterium and Lactobacillus outcomes was found to be low (I²=19% and 39%). The heterogeneity for Bacteroidetes outcomes was found to be high (I²=94%); therefore, a sensitivity analysis was conducted by Stata software to assess the robustness of each included trial for the Bacteroidetes outcome. It was speculated that the study by LI Yan-can 2019 (43) was the overwhelming factor influencing the overall Bacteroidetes outcome, as shown in **Figure 3A**. After eliminating the study by LI Yan-can 2019, the heterogeneity for Bacteroidetes decreased to 0%, so a fixed effects model was utilized to perform the meta-analysis for the 3 outcomes.

**Figure 4:**
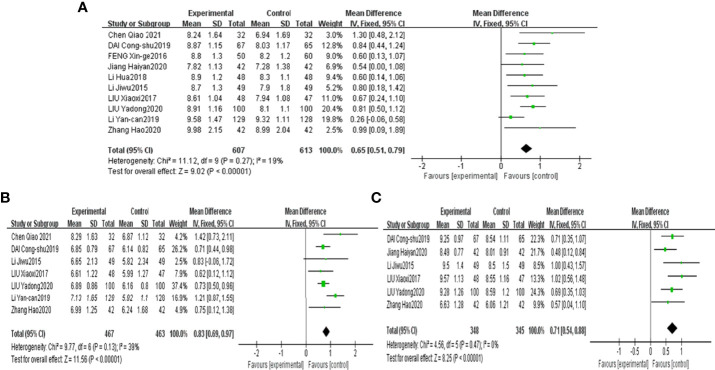
Forest plots of Bifidobacterium **(A)**, Lactobacillus **(B)** and Bacteroidetes **(C)** for the comparison of CHF+metformin treatment versus metformin monotherapy.

The results demonstrated that combined CHF with metformin intervention contributed to a more significant abundance of Bifidobacterium, Lactobacillus and Bacteroidetes than metformin monotherapy, with the results being significantly different [MD=0.65, 95% CI (0.51, 0.79), P<0.00001], [MD=0.83, 95% CI (0.69, 0.97), P<0.00001], [MD=0.71, 95% CI (0.54, 0.88), p<0.00001], as shown in **Figures 4A–C**, respectively.

#### 3.4.2 Opportunistic pathogenic bacteria

To compare the effects of combined CHF and metformin treatment with those of metformin monotherapy on the quantitative changes in opportunistic pathogenic bacteria, 9 studies (36, 39–43, 45–47) describing the changes in Enterobacteriaceae, 8 studies (36, 37, 39–43, 47) describing the changes in Enterococcus and 6 studies (39–43, 47) describing the changes in Saccharomyces were included in the meta-analysis.

The heterogeneity for Enterococcus was found to be moderate (I²=59%); therefore, a random effect model was used to analyse the outcome. The heterogeneity for Enterobacteriaceae and Saccharomyces were found to be high (I²=86% and I²=98%). The sensitivity analyses suggested that the study by LI Yan-can 2019 obviously influenced the overall combined Enterobacteriaceae and Saccharomyces outcomes, as shown in **Figures 3B, C**. After eliminating the study by LI Yan-can 2019 (43), the I² of Enterobacteriaceae and Saccharomyces decreased to 40% and 70%, respectively.

**Figure 5:**
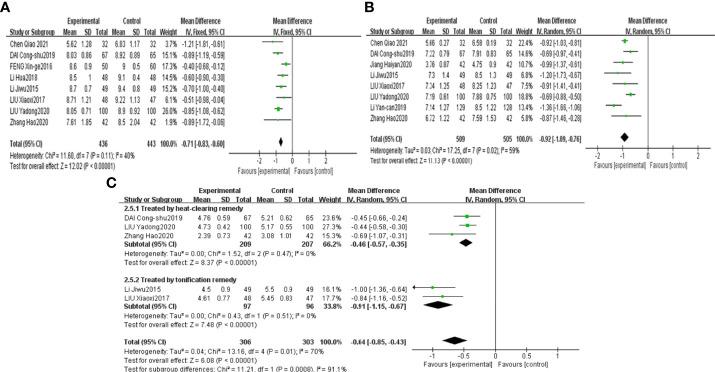
Forest plots of Enterobacteriaceae **(A)**, Enterococcus **(B)** and Saccharomyces **(C)** for the comparison of CHF+metformin treatment versus metformin monotherapy.

The results of the meta-analysis revealed that combined CHF and metformin treatment led to a lower abundance of Enterobacteriaceae, Enterococcus and Saccharomyces than metformin, with the results being significantly different [MD=-0.71, 95% CI (-0.83, -0.60), P<0.00001], [MD=-0.92, 95% CI (-1.09, -0.76), p<0.00001], and [MD=-0.64, 95% CI (-0.85, -0.43), p<0.00001], as shown in **Figures 5A–C**, respectively.

#### 3.4.3 Changes in glucose parameters

To compare the effects of combined CHF and metformin treatment with those of metformin monotherapy on the changes in glucose parameters, 8 studies (36, 38, 40–42, 44–46) comparing the changes in FPG, 7 studies (36, 38, 40, 42, 44–46) comparing the changes in 2hPG, 6 studies (37, 38, 42, 44–46) comparing the changes in HbA1c, 5 studies comparing the changes in FINS (40–42, 44, 47) and 4 studies comparing the changes in HOMA-IR (40–42, 47) were included in the analyses.

The heterogeneity for the six outcomes was high. According to the sensitivity analyses of the six outcomes, the influence of individual studies on the overall outcome was stable, as shown in **Figures 3D–H**.

**Figure 6:**
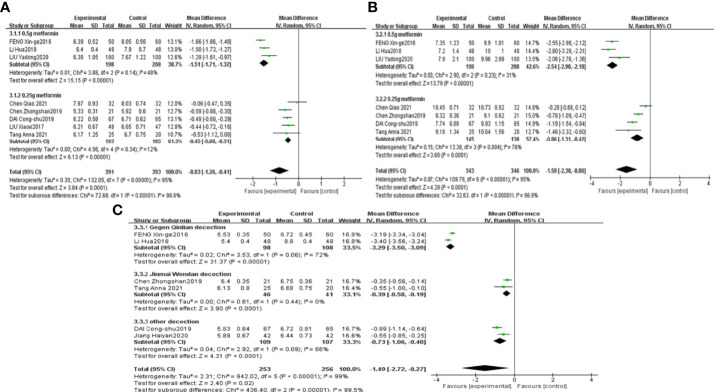
Forest plots of FPG **(A)**, 2hPG **(B)**, HbA1c **(C)** for the comparison of CHF+metformin treatment versus metformin monotherapy.

**Figure 7:**
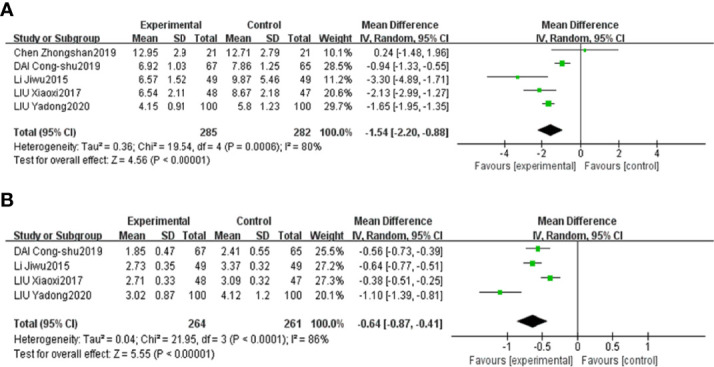
Forest plots of FINS **(A)** and HOMA-IR **(B)** for the comparison of CHF+metformin treatment versus metformin monotherapy.

The results of the meta-analyses confirmed that combined CHF and metformin treatment contributed to remarkably lower levels of FPG, 2hPG, HbA1c, FINS and HOMA-IR than metformin alone [MD=-0.83, 95% CI (-1.26, -0.41), p<0.00001], [MD=-1.58, 95% CI (-2.30, -0.86), p<0.00001], [MD=-1.49, 95% CI (-2.72, -0.27), p<0.00001], [MD=-1.54, 95% CI (-2.20, -0.88), p<0.00001], and [MD=-0.64, 95% CI (-0.87, -0.41), p<0.00001], as shown in **Figures 6A–C**, **Figures 7A, B**, respectively.

### 3.5 Subgroup analyses

Sensitivity analyses showed that the overall results for Saccharomyces, FPG, 2hPG, HbA1c, FINS and HOMA-IR were stable. Only 5 studies and 4 studies individually analysed the changes in FINS and HOMA-IR, respectively; therefore, an inadequate sample size might be the source of the heterogeneity for FINS and HOMA-IR. Thus, subgroup analyses were conducted for Saccharomyces, FPG, 2hPG, and HbA1c to further verify the source of heterogeneity.

Saccharomyces and HbA1c results were grouped by the therapeutic principal classification of the CHFs. The results of 3 trials involving CHFs with the therapeutic principle of heat clearing (39, 40, 42) illustrated that Saccharomyces in the combined treatment group was notably lower than that in the metformin treatment group [MD=-0.46, 95% CI (-0.57, -0.35), P<0.00001]. The results of 2 studies involving CHFs with the therapeutic principal of “tonification” (41, 47) suggested that combination treatment contributed to a noteworthy lower level of Saccharomyces than metformin treatment [MD=-0.91, 95% CI (-1.15, -0.67), P<0.00001], as shown in **Figure 5C**. The results of studies (45, 46) using Gegen Qinlian decoction revealed that the level of HbA1c in the combined treatment group was notably lower than that in the metformin treatment group [MD=-3.29, 95% CI (-3.50, -3.09), p<0.00001]. The results of 2 studies (38, 44) using Jinmai Wendan decoction illustrated that the combined treatment led to a lower level of HbA1c than metformin treatment [MD=-0.39, 95% CI (-0.58, -0.19), p<0.0001]. The results of 2 other studies (37, 42) that used other CHFs revealed that combined treatment contributed to a notably lower level of HbA1c than metformin treatment [MD=-0.73, 95% CI (-1.06, -0.40), p<0.0001], as shown in **Figure 6C**.

FPG and 2hPG studies were grouped according to the doses of metformin administered to investigate the effect of different doses of metformin on glucose metabolism. The results of 3 studies (40, 45, 46) using 0.5 g metformin revealed that combined treatment contributed to a lower level of FPG and 2hPG than metformin treatment, [MD=-1.51, 95% CI (-1.71, -1.32), p<0.00001] and [MD=-2.54, 95% CI (-2.90, -2.18), p<0.00001], respectively. The results of 5 studies (36, 38, 41, 42, 44) using 0.25 g metformin confirmed that FPG in the combined treatment group was notably lower than that in the metformin treatment group [MD=-0.45, 95% CI (-0.60, -0.31), p<0.00001]. The results of 4 studies (36, 38, 42, 44) using 0.25 g metformin also confirmed that the 2hPG in the combined treatment group was notably lower than that in the metformin treatment group [MD=-0.86, 95% CI (-1.31, -0.42), p=0.0001], as shown in **Figures 6A, B**.

### 3.6 Publication bias analysis

**Figure 8:**
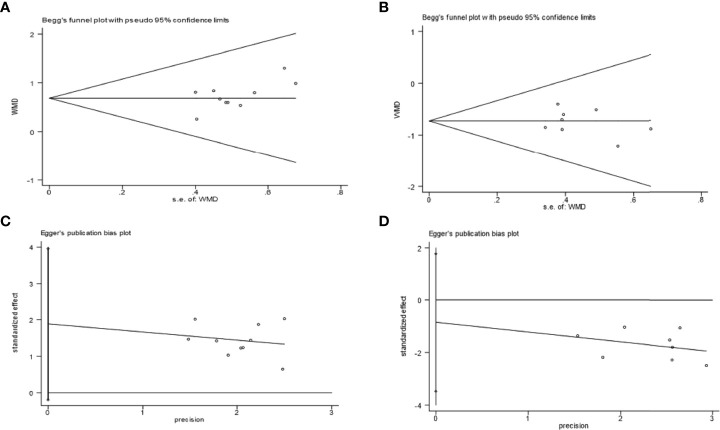
Examples of Begg’s funnel plots: Bifidobacterium **(A)** and Enterobacteriaceae **(B)**; examples of Egger’s publication bias plots: Bifidobacterium **(C)** and Enterobacteriaceae **(D)**.

Taking Bifidobacterium and Enterobacteriaceae as examples, it was observed that the included trials showed balanced distributions in Begg’s funnel and Egger’s publication bias plots, as shown in **Figures 8A–D**. Combined with analysing the data of Pr > |z| in Begg’s tests and Pr > |t| in Egger’s tests, which both tested > 0.05, it was found that there was no significant publication bias in the analysed outcomes in these studies. The data of Pr > |z| and Pr > |t| are summarized in **Table 5**.

**Table 5:** Summary of data of Pr > |z| and Pr > |t| in Begg’s test and Egger’s test.

Outcomes	Begg's test (Pr > |z|)	Egger's test (Pr > |t|)
Bifidobacterium	0.371	0.07
Lactobacillus	0.368	0.533
Bacteroidetes	0.707	0.319
Enterobacteriaceae	0.711	0.458
Enterococcus	0.711	0.498
Saccharomycetes	0.211	0.061
FPG	0.536	0.24
2hPG	0.368	0.372
HbA1c	0.707	0.07
FINS	1	0.838
HOMA-IR	0.308	0.135

## 4 Discussion

In recent years, new lines of evidence have shown the vital role of the gut microbiota in the pathophysiology of diabetes. The mutual relationship between the gut microbiota and glucose metabolism is mainly associated with changes in intestinal permeability, bile acid metabolism, serum lipopolysaccharide (LPS) levels and short-chain fatty acid (SCFA) production (48). The specific mechanisms are as follows: (*i*) The alterations in the gut microbiota in T2DM, which reduce key functional proteins in intestinal epithelial cells and increase intestinal mucosal permeability to promote the release of LPS, followed by the generation of inflammatory cytokines, can decrease the insulin sensitivity of the body as a consequence (49). (*ii*) The increased synthesis of intestinal branched-chain amino acids in line with the elevated concentration of branched-chain amino acids in the circulation is a powerful biomarker of insulin resistance and has the potential to increase the onset risk of T2DM (50), suggesting that changes in the synthesis and concentration of branched-chain amino acids may be a bridge between the gut microbiota and the onset of T2DM. (*iii*) Single-chain fatty acids (SCFAs) might be one of the bridges between the gut microbiota and T2DM. The gut microbiota can decompose carbohydrates transformed from intestinal food into SCFAs, which can regulate the inflammatory response and insulin sensitivity by stimulating the secretion of glucagon-like peptide-1 (GLP-1), accompanied by promoting liver lipid metabolism to maintain glucose homeostasis (51). (*iv*) The concentration and composition of bile acids were found to be modified in T2DM patients. The mutual interaction between the gut microbiota and bile acids can influence the secretion of GLP-1, which contributes to regulating the dynamic balance of glucose metabolism (52). (*v*) It is believed that the gut microbiota also has a potential impact on the immune system to varying degrees. Reports have revealed that when intestinal bacteria are introduced into aseptic mice, the function of the immune system is restored by triggering an immune response, which can further promote the metabolic function of the body (53). These results highlight that the gut microbiota can serve as a novel intervention target in the treatment of diabetes.

In treating type 2 diabetes, Chinese herbal formulas, as one of the important constituents in traditional Chinese medicine (TCM), are now used as a potential adjuvant therapy for treating various diseases by modulating gut microbiota and promoting host metabolism (54–57). Drugs with oral administration can promote the host metabolism and modulate the structure of gut microbiota (58). In term, the gut microbiota could mediate the metabolism of bioactive constituents in oral drugs *via* various targets (59). Previous studies further provided the reliable and direct evidence for the additive and synergistic effects of CHFs and metformin on modulating gut microbiota and ameliorating metabolic disorders in type 2 diabetic patients (60–62). A multicenter, randomized, open label clinical trial has showed that metformin and CHFs changed the gut microbiota composition of T2DM patients. Experiments *in vivo* and *in vitro* have validated that Huangkui capsules combined with metformin treatment effectively improved the weight, reduced blood glucose and ameliorated renal fibrosis *via* the Klotho/TGF-β1/p38 MAPK signalling pathway (28). Shin NR (63) mentioned that the Flos Lonicera, a bioactive component in CHFs, could modulate the gut microbial composition by reverting LPS-related pathways when combined with metformin. Wang K (64) also suggested that Houttuynia cordata, also a key component in CHFs, could suppress MCP-1, IL-6, TLR4 accompanied by modulating the abundances of Gram-negative bacteria when combined with metformin. A randomized placebo-controlled double-blind study indicated that metformin combined with Jianyutangkang therapy had a significant antidiabetic effect and improved lipid profiles with less adverse effects (hepatotoxicity or nephrotoxicity) (60). Chen WP (65) demonstrated that Danhong Huayu Koufuye combined with metformin had a preventive and therapeutic effect on diabetic retinopathy by alleviating hyperglycaemia, reducing oxidative stress and improving lipid metabolism. Liu X (66) concluded that Huoxue Jiangtang decoction combined with metformin could significantly lower blood glucose and serum cholesterol levels and exerted protective effects on kidney tissue damage. Lian F (67) showed that Jinlida combined with metformin significantly improved β-cell function compared to metformin monotherapy. Cao Y (5) showed that Fuzhu jiangtang granules combined with metformin had beneficial effects on lowering FBG, alleviating insulin resistance and restoring glucose tolerance in diabetic rats, possibly by regulating the PI3K/Akt signalling pathway in skeletal muscle. Han K (68) reported that bile acids promoting gut microbiota, such as Lactobacillus and Bacteroides, were higher in the combined treatment group than that in the metformin treatment group. Positive changes in the gut microbiota and host metabolism observed in the CHFs combined with metformin treatment further confirmed the valuable application of the combination treatment. Thus, the application of CHFs combined with metformin therapy has a considerable development prospect for the treatment of type 2 diabetes through the modulation of gut microbiota.

In this study, sensitivity analyses and subgroup analyses identified the sources of the heterogeneity. Sensitivity analyses showed that the overall results of the three outcomes, Bacteroidetes, Enterobacteriaceae and Saccharomyces, were interfered by the study LI Yan-can 2019 (43). The elimination of this study decreased the heterogeneity of the three outcomes to varying degrees (low or modest). The heterogeneity of this study is probably associated with inconsistency in sample size, drug dosage, treatment course, massage time and operation method (69). Then, subgroup analyses found that using 0.5 g or 0.25 g metformin and using CHFs with different therapeutic classifications exerted different effects on T2DM patients, which further suggested the sources of the heterogeneity.

First, different does of metformin influence the changes in FPG and 2hPG differently. Previous studies suggested that the efficacy of the hypoglycaemic activity of metformin was dose dependent (70). Metformin, a widely prescribed oral hypoglycaemic agent that has been used as the first-line treatment option for years (71), can facilitate better glucose homeostasis for diabetes patients through pleiotropic mechanisms (72). Metformin acts predominantly on the liver to inhibit neoglucogenesis and accelerate the muscle intake of glucose (73). Metformin also activates adenosine 5’-monophosphate-activated protein kinase (AMPK), inhibits the mitochondrial respiratory chain and improves insulin sensitivity (74). The recommended dose of metformin ranges from 250 mg/day to over 2000 mg/day in European and Asian countries in different tables of metformin dosing (75, 76). Studies have found that higher doses of metformin induced modestly greater levels of glycaemic control and greater weight and BMI reduction in metabolism-syndrome-related diseases (77). The findings from another randomized controlled trial revealed that metformin may suppress the formation of human colorectal ACF for colorectal cancer patients with IGT in a dose-dependent manner (250, 500, or 1500 mg/day) (78). Chen W (79) concluded that metformin exerted a positive effect by reducing inflammatory responses in a dose-dependent manner in patients with type 2 diabetes.

Second, the therapeutic classifications of CHFs might influence their effects on glucose metabolism and the gut microbiota. In this study, CHFs belonging to the classification of “heat-clearing” remedy included Gegen Qinlian decoction, Jinmai Wentan decoction, Qingfei Xiegan decoction, and Qingre Huoxue Huatan formula. The most frequently reported “heat-clearing” CHF in treating T2DM is Gegen Qinlian decoction (GQD), which was originally used to treat bacterial diarrhoea (80), and GQD was reported to alleviate the symptoms of T2DM in current studies (56). R. Li (81) showed that GQD inhibits the inflammatory signalling pathway and enhances antioxidant effects. C. H (82) proved that GQD plays a positive role in improving glucose metabolism by increasing the insulin sensitivity index for T2DM patients. Huang ZQ (83) found that GQD has a dose-effect relationship with controlling FBG levels in 2-DM model rats, providing new insight into the evaluation of the dose-effect relationship of TCMs. In addition, CHFs belonging to the therapeutic classification of “tonification” remedy included Shenling Baizhu powder, Tonifying Qi and strengthening spleen decoction, and Wenyang Yiqi Huoxue formula. Shenling Baizhu powder (SBP), as a representative Pi-tonifying prescription, is used to treat the syndrome of Pi-deficient diarrhoea. Xiao Y’s research (84) evaluated the underlying mechanism of SBP against Pi-deficient diarrhoea and found that high-dose SBP can modulate the absorption function of the intestine and the immunity function of intestinal mesenteric lymph nodes. Wang X (85) indicated that SBP could significantly inhibit virus replication and proliferation by regulating the TLR4/MyD88/NF-κB signalling pathway. SBP was also administered as an adjuvant therapy to reduce the expression level of serum TH1 cytokines, reduce the inflammatory response, and improve the clinical efficacy in ulcerative colitis patients (86). Tang K (57) found that SBP exerts a positive effect by ameliorating insulin resistance and correcting lipid metabolism disorders.

Moreover, identifying the key components in CHFs for the improvement of T2DM may tremendously promote our understanding of the mechanisms by CHFs acting against diabetes. Cui L (87) validated that oxidative and inflammation stress can be alleviated by some active components in Gegen Qinlian decoction(GQD), such as baicalin, glabridin and berberine, which were identified as the potential bioactive compounds of GQD in treating T2DM. Berberine, which was hypothesized as the key pharmaceutical ingredient in GQD, was reported to significantly alleviate hyperglycaemia and alter gut microbiota structure as GQD did (56, 88) by preventing and alleviating obesity and insulin resistance (69). An integrated system pharmacology analysis then revealed that various targets such as PPARG, RELA, EGFR and numerous pathways such as TNF signalling, PI3K-Akt signalling, MAPK signalling and NF-κB signalling were involved in the underlying mechanisms of GQD in improving diabetic insulin resistance (89). In GQD, berberine can enhance the preferable antidiabetic activity of metformin by modulating the distribution of metformin in different organs (90). Zhang X (91) illustrated the mechanisms by which berberine prevented obesity and insulin resistance by reducing opportunistic pathogens and enriching short-chain-fatty-acid-synthesizing bacteria. H. Zhou (92) showed that berberine can activate AMPK-dependent autophagy to reduce inflammation and insulin resistance. L. Ye (93) indicated that berberine improved insulin resistance possibly through inhibiting the activation of macrophages in adipose tissue. Another typical CHF used in treating diabetes is Shenling Baizhu powder(SBP). In Shenling Baizhu powder, Baizhu which contained the inulin-type oligosaccharides, was thought as a key component in SBP to improve the abundance of SCFAs-generating gut microbiota (94). Thus, alleviating inflammation stress and shifting the gut microbiota by targeting certain pathways and signals may be the mechanisms of bioactive constituents, such as berberine and Baizhu in GQD and SBP, to alleviate insulin resistance and reshape host metabolism.

However, inadequate studies have compared and analysed the mechanism of how metformin and CHFs in different forms influence hyperglycaemia and gut microbiota differently. Thus, we aimed to explore the mechanism of metformin doses and CHF classifications on controlling hyperglycaemia and altering gut microbiota. Moreover, although the publication bias analyses did not show significant bias in these analysed studies, some limitations of this study should be pointed out. First, the methodological designs of many of the included studies still have flaws; therefore, future studies of higher methodological quality are needed. Second, the direct pharmacological mechanisms studies of the CHFs are meeting difficulties because of the diverse components of CHFs and complex interactions between constituents through physical and chemical reaction (95). Therefore, more well-designed RCTs with objective methods are needed to elucidate and investigate the effects of each component on enhancing the structure of the gut microbiota in the treatment of diabetes.

## 5 Conclusion

In conclusion, the results of this meta-analysis are inspiring and provide evidence supporting the potential effectiveness and safety of combined CHF and metformin therapy in controlling hyperglycemia and regulating the gut microbiota. Despite some limitations, the results contribute to supporting the value of combined CHF and metformin treatment in diabetes patients. Moreover, more well-designed RCTs are needed to explore the underlying mechanism of this combination treatment and to verify the treatment value in the amelioration of type 2 diabetes mellitus.

## Data availability statement

The original contributions presented in the study are included in the article/supplementary material. Further inquiries can be directed to the corresponding author.

## Author contributions

YX and YZ designed the study and were in charge of the study concept. SJ and JC searched online databases and screened the eligible studies. YX and SZ extracted data and collected the data. SZ and XZ evaluated the methodological quality of each included study. YX interpreted the results and wrote the manuscript. YZ resolved disagreements, revised the manuscript and provided funding. All authors reviewed the manuscript.

## Funding

This research was supported by the Sichuan Provincial Administration of Traditional Chinese Medicine (Grant Number: 2021MS457).

## Acknowledgments

I would like to acknowledge all of my team members who provided a great deal of support and assistance to fulfil this research.

## Conflict of interest

The authors declare that the research was conducted in the absence of any commercial or financial relationships that could be construed as a potential conflict of interest.

## Publisher’s note

All claims expressed in this article are solely those of the authors and do not necessarily represent those of their affiliated organizations, or those of the publisher, the editors and the reviewers. Any product that may be evaluated in this article, or claim that may be made by its manufacturer, is not guaranteed or endorsed by the publisher.

## Abbreviations

T2DM, type 2 diabetes mellitus; TCM, traditional Chinese medicine; FPG, fasting plasma glucose; 2hPG, 2-hour postprandial blood glucose; HbA1c, glycated haemoglobin; FINS, fasting insulin; HOMA-IR, homeostasis model assessment of insulin resistance; NMPN, national medicine permission number.
